# A Comprehensive Biochemical Characterization of Hybrid Grouper Larvae (*Epinephelus fuscoguttatus*♀ × *Epinephelus lanceolatus*♂) during Yolk-Sac Larval Development

**DOI:** 10.3390/ani13243801

**Published:** 2023-12-09

**Authors:** Xiaoqiang Gao, Shuquan Cao, Rongjie Chen, Fan Fei, Wenyang Li, Xianhong Zhang, Zhiwen Zhu, Baoliang Liu

**Affiliations:** 1 State Key Laboratory of Mariculture Biobreeding and Sustainable Goods, Yellow Sea Fisheries Research Institute, Chinese Academy of Fishery Sciences, Qingdao 266071, China; gaoxq@ysfri.ac.cn (X.G.); w6057176@163.com (S.C.); feif_1122@163.com (F.F.); rose-hwsh@163.com (W.L.); qd2023zxh@163.com (X.Z.); w19731224@sina.com (Z.Z.); 2 Fisheries College, Ocean University of China, Qingdao 266100, China; 3 Laizhou Marine Development and Fisheries Service Center, Yantai 261400, China; miqinger@126.com

**Keywords:** hybrid grouper, development stage, lipid, protein, fatty acid, amino acid

## Abstract

**Simple Summary:**

This study assessed changes in the biochemical composition of hybrid grouper during the early larval stages. Samples were collected at various developmental stages, ranging from newly hatched larvae to 4 days after hatching. The findings reveal several key observations: (1) The total length of hybrid grouper larvae exhibited a significant increase as the yolk-sac absorption progressed from stage I to V. (2) Dry weight and total lipid content exhibited a rapid decrease throughout larval development. (3) Significant reductions were observed in the levels of triacylglycerols and wax esters/steryl esters. (4) Throughout yolk-sac larval development, a notable decrease occurred in the concentrations of essential amino acids, including leucine, valine, isoleucine, phenylalanine, glycine, alanine, serine, proline, and tyrosine. (5) There was a significant decrease in the levels of specific fatty acids, such as C16:0, saturated fatty acids (SFAa), monounsaturated fatty acids (MUFAs), C18:0, 18:1n-9, and C20:4n-6. Conversely, the levels of C22:6n-3, polyunsaturated fatty acids (PUFAs), n-3 PUFA, n-6 PUFA, and the combination of docosahexaenoic acid (DHA) + eicosapentaenoic acid (EPA), as well as the DHA/EPA ratio, remained stable from stage I to III but decreased thereafter. (6) During the early developmental stages, the utilization sequence of fatty acids followed a pattern of SFAs, MUFAs, n-6 PUFA, and n-3 PUFA. These results provide further insights into the varying efficiency of utilization among different types of fatty acids and highlight relatively stable protein utilization with selective consumption of amino acid content during hybrid grouper larval development.

**Abstract:**

To investigate the shifts in the biochemical composition of hybrid grouper during the early larval stages, we collected samples at various developmental milestones, spanning from newly hatched larvae (stage I) to 4 days after hatching (stage V). Our findings revealed several notable trends: (1) The total length of hybrid grouper larvae exhibited a significant increase as the yolk-sac absorption progressed from stage I to V. Concurrently, there was a marked decrease in yolk volume and oil volume during the transition from stage I to III, followed by a gradual decline from stage III to V. (2) Dry weight and total lipid content displayed a rapid reduction throughout the larval development period, while the total protein content exhibited a declining trend. (3) The concentrations of triacylglycerols and wax esters/steryl esters decreased considerably, particularly at stage V. However, no differences were observed among the contents of ketones, hydrocarbons, and sterols. (4) As yolk-sac larvae developed from stage I to V, a significant reduction was observed in the levels of essential amino acids (EAAs), such as leucine, valine, isoleucine, phenylalanine, glycine, alanine, serine, proline, and tyrosine. This trend was also observed for non-EAAs and total amino acids, with fluctuations in the content of other amino acids. (5) There was a significant decrease in the levels of specific fatty acids, including C16:0, saturated fatty acids (SFAs), monounsaturated fatty acids (MUFAs), C18:0, 18:1n-9, and C20:4n-6. In contrast, the contents of C22:6n-3, polyunsaturated fatty acids (PUFAs), n-3 PUFA, n-6 PUFA, and the combination of docosahexaenoic acid (DHA) + eicosapentaenoic acid (EPA), as well as the DHA/EPA ratio, remained stable from stage I to III but declined thereafter. (6) During the early developmental stages, the utilization sequence of fatty acids followed a pattern of prioritizing SFAs, followed by MUFAs, n-6 PUFA, and n-3 PUFA. These findings provide further insights into the nutritional priorities of hybrid grouper larvae during their early development, with a particular emphasis on lipids and fatty acids as vital energy sources. Additionally, our results highlight variations in the efficiency of utilization among different types of fatty acids, while protein utilization remained relatively stable, characterized by the selective consumption of amino acid content.

## 1. Introduction

The pearl gentian grouper, scientifically known as *Epinephelus fuscoguttatus*♀ × *Epinephelus lanceolatus*♂ and commonly referred to as the “tiger grouper” or “pearl grouper”, has gained significant prominence in the aquaculture industry. Renowned for its desirable attributes and affordability, the pearl gentian grouper has emerged as a highly sought-after commercial fish, often hailed as the “rising star” of the grouper sector [1]. In China, the farming methods for pearl gentian grouper have evolved, encompassing successful approaches, such as cage and pond farming. However, one notable development is the increasing popularity of indoor factory farming, with a particular emphasis on the application of recirculating aquaculture systems [2]. Despite these advancements, the sustainable growth of the grouper industry faces various challenges. These include genetic resource deterioration, low hatchery survival rates, and elevated mortality rates, particularly during the critical mouth-opening stage. High mortality during the embryonic and yolk-sac stages can significantly impede hatchery production [3,4,5]. Guo et al. [6] have suggested that the elevated mortality of early-stage larvae during the transition from endogenous to exogenous nutrition may be linked to larval quality.

During the yolk nutrition phase, before the onset of exogenous nutrition intake, fish embryos and early larvae rely primarily on endogenous substances stored within the yolk for their nutritional needs. The major components found in the yolk, including proteins and amino acids, lipids and fatty acids, and carbohydrates, constitute the foundational building blocks for embryonic and early larval development in fish. These components play a pivotal role not only during this phase but also in subsequent stages, including the transition to exogenous feeding, fry and juvenile development, and overall survival [7,8,9]. The consumption sequence of endogenous nutrient components is helpful to understand the dynamic changes of nutrient utilization in larva at yolk sac stage [10,11]. It is noteworthy that the biochemical composition of yolk-sac larvae can vary among species and may undergo changes during different developmental stages due to environmental conditions, physiological events, and energy demands [12,13,14]. Amino acids, in particular, play a significant role in structurally shaping embryonic and early larval development [15,16,17], while lipids serve as primary energy sources and contribute to tissue formation and physiological regulation during the early developmental stages of fish [18]. Among fatty acids, highly unsaturated fatty acids of the n-3 series, such as docosahexaenoic acid (DHA) and eicosapentaenoic acid (EPA), are essential for the larvae and juveniles of marine species, influencing growth and physiological functions [19,20]. Larval nutrition holds a pivotal position in aquaculture, as it profoundly influences larval survival and growth.

This study represents a pioneering effort, encompassing a comprehensive biochemical analysis of yolk-sac larvae samples. It focused on assessing the dynamic changes of conventional nutritional components, amino acids, and fatty acids during the pre-mouth-opening endogenous nutrition phase. The objective was to gain insights into the actual utilization levels of nutrition during the early developmental stages of the hybrid grouper. The results add considerably to the knowledge of early leptocephalus nutritional information of the hybrid grouper.

## 2. Materials and Methods

### 2.1. Raising Conditions

A total of 500 g of high-quality fertilized hybrid grouper eggs were procured from the Ledongheng Aquatic Group, Haikou, Hainan, China. Subsequently, these eggs were transferred to a closed recirculation aquaculture system (RAS) consisting of four circular ponds, each with a diameter of 2 m and an effective depth of 1 m. Each pond accommodated 125 g of fertilized eggs, and the experiment was conducted with four replicates. Water parameters, including temperature, dissolved oxygen, salinity, and pH, were monitored twice daily (morning and evening) using a digital multiparameter controller (DIQ/S 182XT-4, Tianjin, China). The nitrite concentration was determined spectrophotometrically using the Griess method, while total ammonia nitrogen content was estimated via nesslerization, with NH4Cl as the standard solution. Throughout the incubation period, the mean water conditions were maintained as follows: water temperature, 28.2 ± 0.6 °C; dissolved oxygen, 7.14 ± 0.65 mg/L; pH, 7.1 ± 0.1; salinity, 31.0 ± 1.0 ppt; total ammonia, 0.12 ± 0.02 mg/L; and nitrite, 0.15 ± 0.02 mg/L. The water flow rate within each pond was set to achieve 100% exchange every 6 h, renewing approximately 1% of the RAS overall daily water volume. The photoperiod was maintained at 12 h of light followed by 12 h of darkness.

### 2.2. Sampling and Design

After hatching, 800 mg (*n* = 1000) of larvae were systematically collected from each circular pond continuously from the moment of hatching (0 days after hatching; DAH) until 4 DAH. The sampled larval developmental stages encompassed hatching (stage I), the fast yolk-sac absorption/growing stage at 1 DAH (stage II), the yolk-sac exhaustion stage at 2 DAH (stage III), the mouth-opening starvation stage at 3 DAH (stage IV), and the starvation-weakened stage at 4 DAH (stage V). All collected samples were promptly frozen in liquid nitrogen and stored at –80 °C until biochemical analysis. 

For morphometric analysis, a subsample of 15 larvae was collected daily from each circular pond. These larvae were photographed using the digital camera Moticam Pro 28 mounted on a Leica DM2500 stereomicroscope (Tonghao Photoelectric Technology Co., Ltd., Shanghai, China). Measurements were carried out using ImageJ software (V1.8.0.112), with linear measurements obtained parallel to the longitudinal axis of the larvae body [21]. Morphometric formulae were calculated following the descriptionby Khemis et al. [22]. The total length (TL) of each larva was measured from snout to tail tip. The volume of the yolk was estimated using the volumetric formula for an ellipsoid: V = (π/6) LH^2^, where L corresponded to the major axis and H corresponded to the minor axis of the yolk. The volume of the oil globule was determined using the volumetric formula of a sphere, with the diameter measurement used to derive the radius.

### 2.3. Proximate Composition Analysis

The larval samples underwent analysis based on the standard methods outlined in the AOAC (2000) procedure. The dry matter content was determined gravimetrically by weighing the sample once it reached a constant weight after drying in an oven at 105 °C. Moisture content was calculated from the weight difference in specimens dried for 24 h in an oven at 105 °C. Ash content was measured by incinerating the sample in a muffle furnace (SX-4-10, Beijing Ever Bright Medical Treatment Instrument Co., Ltd., Beijing, China) at 600 °C for 4 h. Crude protein content was determined using the Kjeldahl method (N × 6.25) via an automatic Kjeldahl nitrogen analyzer (Jinan Olaibo Equipment Co., Ltd., OLB9870, Jinan, Shandong, China). Crude lipid content was quantified through petroleum ether extraction using Soxhlet (Buchi 36680, Flawil, Switzerland). Lipid class composition and profiles of the larval samples were determined following methods developed by Parrish [23]. Flame ionization detection techniques via Dionex™ ICS-6000 (Thermo Scientific, Shanghai, China) were employed for lipid class composition analysis. The resulting chromatograms facilitated the identification of various lipid classes, including triacylglycerols (TAGs), wax esters/steryl esters (WE-SE), ketones (KET), hydrocarbons (HC), sterols (ST), and phospholipids (PL).

### 2.4. Fatty Acid Analysis

Total lipids were individually extracted using chloroform-methanol (2:1 *v*/*v*) containing 0.01% butylated hydroxytoluene as an antioxidant, in line with the methods of Folch et al. (1957) [24]. Fatty acid methyl esters (FAMEs) were prepared by transesterification with boron trifluoride in methanol, following the techniques outlined by Liu et al. [25]. FAMEs were detected as per the methodsof Gao et al. [26]. These FAMEs were then separated and quantified using a chromatograph (GS, HP6890, Hewlett-Packard, Palo Alto, CA, USA) equipped with a flame ionization detector (250 °C) and a SPTM-2380 column (30 m × 0.25 mm i.d., CP wax 52CB; Chrompak, London, UK). Hydrogen served as the carrier gas, with temperature programming from 50 °C to 160 °C at 5 °C/min, followed by an increase to 225 °C at 2 °C/min. Individual FAMEs were identified by reference to authentic standards (C4C24 by Supelco; Sigma-Aldrich, St. Louis, MO, USA) and well-characterized fish oil (Mehaden oil by Supelco; Sigma-Aldrich). All measurements were performed in triplicate.

### 2.5. Amino Acids Analysis

Amino acid composition in the larvae was determined using an automatic amino acid analyzer (L-8900, Hitachi, Tokyo, Japan). A 50 mg freeze-dried sample was hydrolyzed in 6 mol/L HCl containing 0.5% phenol at 110 °C for 24 h under an argon atmosphere. After dilution with ultra-clean water, the hydrolysate was transferred to a rotary evaporator flask and evaporated to dryness (75 °C). The samples were then resuspended in 0.1 N HCl loading buffer and filtered through a 0.22 μm polyether sulfone ultrafiltration membrane. Finally, amino acid analysis was conducted using a high-performance liquid chromatography system (1100; Agilent Technologies, Inc., Santa Clara, CA, USA). It is important to note that tryptophan was destroyed during acid hydrolysis and was not measured in this study.

### 2.6. Statistical Analysis

All data underwent tests for normality and homogeneity of variance assumptions using Kolmogorov–Smirnov and Levene’s tests, respectively, before subsequent analysis. Experimental data are presented as the mean ± standard deviation (SD). One-way analysis of variance (ANOVA) was employed for data analysis using IBM SPSS v24.0 software (IBM Corp., Armonk, NY, USA). Duncan’s multiple comparisons test was used to identify differences between the treatment means, with significance set at *p* < 0.05.

## 3. Results

### 3.1. Changes in Larval Growth, Yolk Volume, and Oil Volume during Early Development

**Figure 1:**
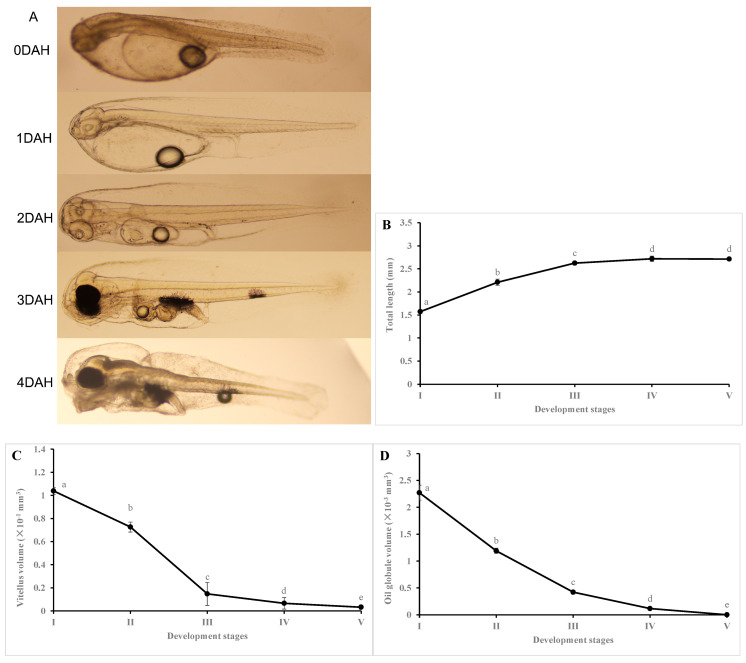
Changes in larval morphology (**A**), total length (**B**), vitellus volume (**C**) and oil globule volume (**D**) in hybrid groupers at different early developmental stages. I, II, III, IV, and V represent groups for newly hatched larvae (0 DAH), 1 DAH, 2 DAH, 3 DAH, and 4 DAH, respectively (*n* = 60). Values are presented as the mean ± SD. Different letters indicate significant differences between groups at *p* < 0.05.

Significant changes in larval growth, yolk volume, and oil volume were observed at various early developmental stages (Figure 1). The TL of hybrid grouper larvae increased significantly as the yolk sac was absorbed from stage I to V (*p <* 0.05), with no differences between stage IV and V (*p* > 0.05). The yolk volume showed a marked decrease when larvae developed from stage I to III, after which a slow drop was observed from stage III to V (*p* < 0.05). Similarly, oil volume displayed a comparable changing pattern from stage I to V (*p* < 0.05).

### 3.2. Changes in Proximate Composition during Early Development of Hybrid Grouper

**Figure 2:**
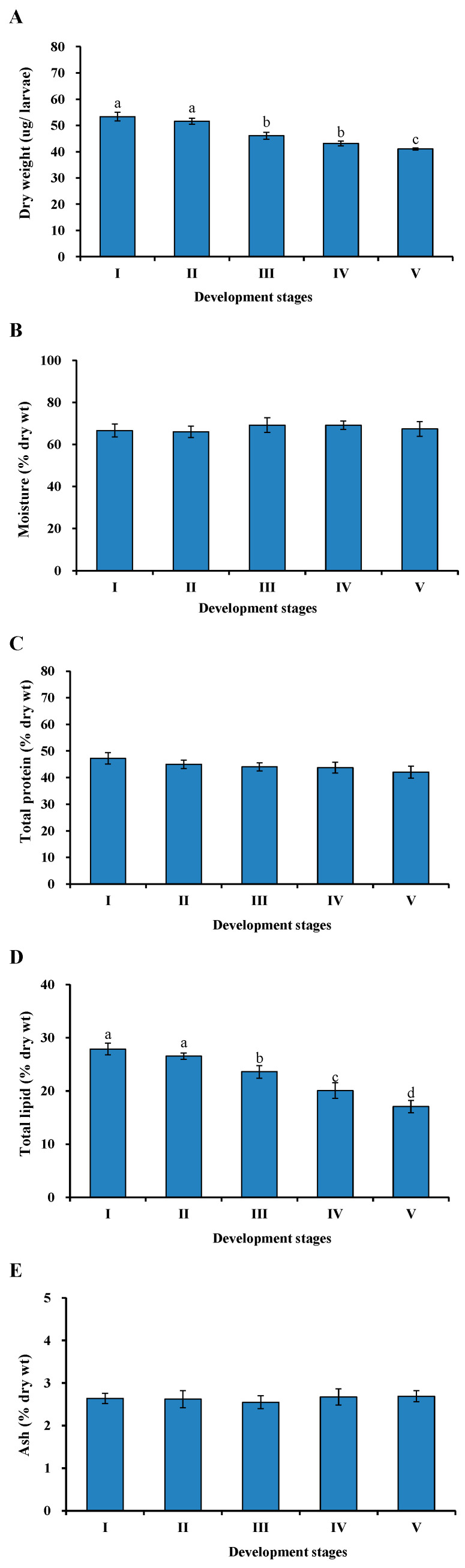
Proximate composition of hybrid grouper larvae at various early developmental stages ((**A**) Dry weight; (**B**) Moisture; (**C**) Total protein; (**D**) Total lipid; (**E**) Ash; *n* = 4000). I, II, III, IV, and V correspond to groups for newly hatched larvae (0 DAH), 1 DAH, 2 DAH, 3 DAH, and 4 DAH, respectively. Values are presented as the mean ± SD. Different letters indicate significant differences between groups at *p* < 0.05.

The results of proximate composition analysis in hybrid grouper larvae at each developmental stage are depicted in Figure 2. The dry weight content displayed a decreasing trend from stage I to IV, with a significant decrease at stage V (*p* < 0.05). Total lipid content exhibited a rapid decrease during larval development (*p* < 0.05). In contrast, total protein, moisture, and ash contents remained unaffected by larval development (*p* > 0.05).

### 3.3. Changes in Lipid Classes during Early Development of Hybrid Grouper

Table 1 displays the lipid classes present in hybrid grouper larvae. The most abundant lipid class was ST, followed by PL, TAG, WE-SE, and HC, with HC being the least abundant. As larvae progressed from stage I to V, the contents of TAG and WE-SE notably decreased, especially in stage V (*p* < 0.05). The PL content remained stable from stage I to III, followed by a significant decrease from stage IV to V (*p* < 0.05). No differences were observed among the contents of KET, HC, and ST (*p* > 0.05). 

### 3.4. Changes in Amino Acid Composition during Early Development of Hybrid Grouper

**Figure 3:**
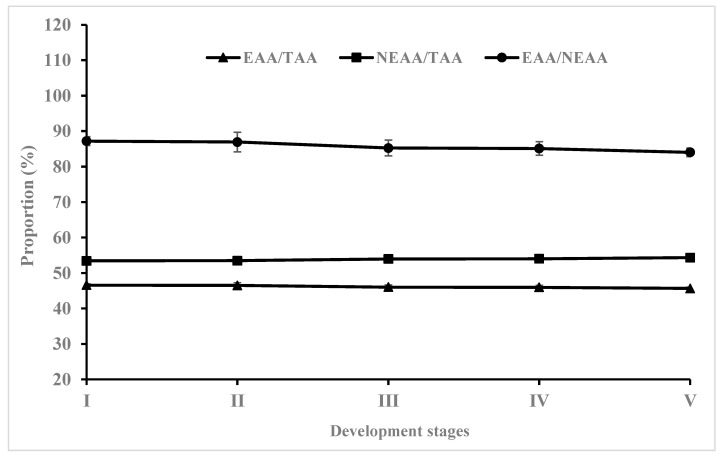
Proportional contents of EAAs and NEAAs in TAAs at different early developmental stages (*n* = 4000). Data are presented as the mean ± SD.

Table 2 presents the whole-body amino acid composition of hybrid grouper larvae. In total, nine essential amino acids (EAAs) and eight non-EAAs (NEAAs) were identified at each stage. During the development of yolk-sac larvae, leucine, lysine, arginine, and phenylalanine were the predominant components in EAAs, while glutamic acid, aspartic acid, alanine, and glycine were the main components in NEAAs. A significant reduction was observed in the contents of leucine, valine, isoleucine, phenylalanine, glycine, alanine, serine, proline, and tyrosine as the larvae progressed from stage I to V (*p* < 0.05). Notably, valine (32.83%), leucine (30.15%), and alanine (26.74%) showed relatively higher losses. Similarly, EAAs, NEAAs, and total amino acids (TAAs) all exhibited a significant decrease in concentration from stage I to V (*p* < 0.05). The contents of other amino acids fluctuated, with no differences (*p* > 0.05). With continuing development, there was no difference in the proportion of EAAs and NEAAs in TAAs among groups (*p* > 0.05). Similar trends were observed in the proportion of EAAs in NEAAs (Figure 3).

### 3.5. Changes in Fatty Acid Composition during Early Development of Hybrid Grouper

The fatty acid composition (% of total) of different developmental stages of hybrid grouper larvae is shown in Table 3. The fatty acids primarily consisted of monounsaturated fatty acids (MUFAs), polyunsaturated fatty acids (PUFAs), and saturated fatty acids (SFAs), including n-3 PUFA and n-6 PUFA. PUFAs had the highest contents, followed by SFAs and MUFAs. During the initial development, the highest fatty acid contents were detected in SFA C16:0, MUFA C18:1n-9, and PUFA C22:6n-3. As the yolk-sac larvae developed, a significant decrease was found in the contents of C16:0, SFAs, MUFAs, C18:0, 18:1n-9, and C20:4n-6 (*p* < 0.05). The contents of C22:6n-3, PUFAs, n-3 PUFAs, n-6 PUFAs, and DHA + EPA, as well as the ratio of DHA/EPA, remained stable from stage I to III, after which they decreased (*p* < 0.05), while a significant increase was detected in the ratio of EPA/ARA (*p* < 0.05).

## 4. Discussion

It remains largely unknown why high mortality is frequently observed in consequential yolk-sac larvae when they undergo the change from endogenous reserves to exogenous feeding. Previous research has suggested that larval sizes at hatching and at mouth opening are key factors affecting early larval survival [22]. This is believed to result from the unavailability of the correct source of food and an underdeveloped digestive system [27]. In our study, as the larvae grew and developed, there was a noticeable decrease in yolk volume and oil volume from stage I to III, followed by a gradual decline from stage III to V. However, the growth of the larvae from stage I to III was rapid, indicating that the critical developmental period for pearl gentian yolk-sac larvae occurs within 0–2 DAH. As the larvae progressed from stage III to V, the yolk-sac and oil globule volume were almost depleted, resulting in slow growth and increased mortality. Additionally, our results showed a significant increase in mortality when the larvae exceeded 4 DAH, suggesting that the transition from endogenous to exogenous nutrition in pearl gentian grouper larvae occurs within a short period. Therefore, providing appropriate and palatable live feed is crucial for ensuring the survival of the larvae.

Embryogenesis is a complex process in teleost fish, heavily reliant on enzymatic systems for yolk utilization, metabolism, energy, and the development of substances [28]. During the yolk-sac larval development stage, the larvae primarily rely on endogenous yolk nutrition, with lipids and proteins serving as the major nutrients. These nutrients provide essential metabolic capabilities and play crucial roles in the synthesis of biological membranes [29,30,31]. Species-specific differences in lipid and protein metabolism have been reported during early fish development [32], indicating distinct utilization preferences among different species. In our study, there was a significant decrease in the total lipid content of the larvae as their dry weight decreased, while the total protein content showed a less pronounced decrease. The decline in the dry matter content of the larvae may be attributed to the substantial consumption of endogenous nutrient reserves. Additionally, a marked decrease in total lipid content during early larval development has been observed in white seabream (*Diplodus sargus*) [33]. Tong et al. [28] also observed a downward trend in lipid content in developing yolk-sac larvae of turbot (*Scophthalmus maximus*), with lipid levels reaching their lowest point at 2 DAH. These findings suggest that lipids may serve as crucial energy sources in the early developmental stages of certain fish species. However, it is worth noting that Liu et al. [34] found stable lipid content in the yolk-sac larvae of American Shad (*Alosa sapidissima*), while the protein and amino acid contents decreased significantly with decreasing dry weight content.In contrast, total lipid content remained constant during the yolk-sac larval development of brill (*Scophthalmus rhombus L*.), with total protein content decreasing during this period, particularly at hatching as observed by Cruzado et al. [12]. Similar patterns have been observed in other fish species, such as barfin flounder (*Verasper moseri*) [35]. These findings suggest that in certain fish species’ early developmental stages, proteins, rather than lipids, serve as the essential energy-providing substance. Furthermore, the research on cobia (*Rachycentron canadum*) by Huang et al. [32] revealed a substantial decrease in the dry weight of larvae during the larval period, while the change in total lipid content did not differ significantly from that of the protein content in 3-day-old larvae. Interestingly, in the case of rainbow trout (*Oncorhynchus mykiss*) larvae, during their development and growth relying on endogenous feeding, the yolk-sac gradually depletes, and body moisture increases to support routine metabolic and catabolic processes, as described by Irani and Noori [36]. However, in the present study, there were no significant changes observed in the moisture content of the larvae. These variations in results indicate that the biochemical composition of yolk reserves in fish is species specific, and the precise sequence of consumption varies both qualitatively and quantitatively, as noted by Liu et al. [37].

The analysis of lipid class profiles in the samples of hybrid grouper yolk-sac larvae revealed the dominant lipid classes to be ST, PL, TAG, and WE-SE. Laurel et al. [38] noted that lipid class profiles change at larval stages, with PL content changing as larvae utilize PL for cellular formation. These lipid classes can be categorized into two groups: structural and storage lipids. PLs play a crucial role as structural components within the lipid bilayer of cells, underscoring their importance in early development for cellular formation. Rainuzzo et al. [39] also emphasized the conservation of PLs across various marine fish larvae species due to their significance in maintaining cell membrane integrity. In this study, the PL content remained stable during the growth and development of yolk-sac larvae but then significantly decreased. This observation suggests that PLs, as essential structural components, are conserved when the yolk sac of pearl gentian grouper larvae is rich in nutrients. However, when there is a substantial depletion of endogenous nutrients, PLs are utilized to a greater extent for energy purposes. Storage lipids can be further categorized into short-term and long-term energy storage lipids, where short-term energy storage lipids like TAG are metabolized before long-term energy storage lipids such as WE-SE [40,41]. In this study, a significant decrease in TAG content was observed during the development of pearl gentian grouper yolk-sac larvae. Conversely, WE-SE content remained relatively stable during stages I and II but exhibited a significant decrease from stage III to V. These findings indicate that storage lipid reserves are gradually depleted during development due to their utilization in metabolic processes related to growth. Furthermore, this suggests that TAG is the primary energy metabolite during the early developmental stages of pearl gentian grouper larvae, followed by WE-SE.

During the development of hybrid grouper yolk-sac larvae, there were distinct trends in amino acid contents. Among the EAAs, the LEU, VAL, ISO, and PHE levels, as well as the NEAA GLY, ALA, SER, PRO, and TYR levels, showed significant decreases. Similar trends have been observed in other species during early developmental stages, with significant reductions in amino acid contents noted by Chang et al. [9] in *Cynoglossus semilaevis* and by Liu et al. [34] during the yolk-sac phase. Additionally, Tong et al. [28] found substantial reductions in almost all amino acid contents during the development of yolk-sac larvae in turbot (*S. maximus*). These findings indicate significant variations in the utilization patterns and selective preferences of amino acids among different fish species during early developmental stages. Similar patterns of selective amino acid utilization have been observed in other species, such as *Odontobutis potamophila*, *Sebastiscus marmoratus*, and *Sebastes schlegelii* [6,11,42]. In this study, it was observed that LEU and VAL, both EAAs, were utilized at relatively high rates during the development of yolk-sac larvae, reaching 30.15% and 32.83%, respectively. Previous studies have highlighted the importance of LEU, as it can be broken down into acetyl-CoA and acetoacetate, crucial intermediates in carbohydrate and lipid metabolism, contributing to energy production through the tricarboxylic acid cycle. LEU also plays a vital role in embryonic tissue and organ formation, as well as cellular regeneration [25,43]. VAL, on the other hand, is involved in carbohydrate metabolism and is considered an energy-producing amino acid. Furthermore, it stimulates muscle development and regeneration [32]. Therefore, the significant decrease in the levels of LEU and VAL during this stage may be associated with their utilization patterns in energy metabolism and growth. 

EAAs play a pivotal role as functional substances and are essential for the early growth and development of fish. Zhu et al. [17] highlighted that the utilization of the FAA pool is not evenly distributed between EAAs and NEAAs. In the yolk-sac larvae of *Hippoglossus hippoglossus*, there is a significant depletion of NEAAs compared to EAAs, particularly with higher consumption rates observed for SER and ALA within the NEAA category. This finding suggests that the EAAs within the FAA pool are preserved primarily for protein synthesis purposes. In the present study, we observed decreases in the total contents of EAAs and NEAAs of 16.41% and 13.25%, respectively, as the yolk-sac larvae of the hybrid grouper developed. However, the ratio of EAAs to NEAAs showed minimal changes, indicating the absence of selective preservation of EAAs during the early developmental stages of pearl gentian grouper. These findings align with a study conducted by Liu et al. [25] on *A. sapidissima*. Additionally, a significant decrease in the total content of FAAs was observed throughout the developmental stages of the larvae, indicating that the depleted FAAs can be used as both energy substrates and precursors for the synthesis of lipids, glucose, proteins, and some other quantitatively unimportant N-rich substances [43].

During the period of endogenous nutrition in fish embryos and early larvae, their life activities entirely rely on the nutrient reserves within the eggs. Fat, as the major energy storage substance in fish eggs, serves not only as a crucial metabolic fuel during early fish development but also participates in organ and tissue formation as well as the regulation of life activities. Therefore, the contents of lipids and fatty acids play a vital role in the development and survival of larvae during the endogenous nutrition phase [44,45,46]. In this study, the main fatty acid composition of yolk-sac larvae of the pearl gentian grouper was found to be highest in C16:0, followed by DHA and C18:1n-9. This result is consistent with findings in *S. marmoratus* [6] and *Takifugu flavidus* [18], indicating the crucial importance of these fatty acids in the early development of pearl gentian grouper larvae. Additionally, in this study, significant decreases in C16:0, C18:0, C20:4n-6 (ARA), and 18:1n-9 were observed from stage I to V of yolk-sac larvae. These findings suggest that C16:0 and C18:0 in SFAs, as well as 18:1n-9 in MUFAs, serve as important energy sources during the yolk-sac stage of pearl gentian grouper development. ARA is an important component of PL and a precursor to eicosanoids. In this study, a significant decrease in ARA during the developmental stages may be attributed to the extensive synthesis of eicosanoids. Studies have shown that substances related to eicosanoids play a significant role in various physiological functions during the early development of fish, particularly in the development, growth, and survival of larval fish immune systems [47].

DHA and EPA are also considered essential fatty acids for human and animal growth and development. The larval stage is a critical period of rapid development for the brain and visual system, requiring significant amounts of DHA and other important nutrients to meet the demands of brain and visual development [26,48,49]. In this study, the content of C22:6n-3 (DHA) remained stable from stage I to III but decreased thereafter. On the other hand, the content of C20:5n-3 (EPA) remained relatively stable throughout the developmental stages. These findings suggest that the yolk-sac larvae of hybrid grouper prioritize the preservation of EPA during early development while consuming a significant amount of DHA. Our results are similar to those of previous studies, such as those investigating *T. flavidus* [18], *S. marmoratus* [6], and *C. semilaevis* [9]. A significant decrease in DHA content further emphasizes the importance of rapid development in the nervous system (brain and retinas) during early fish development. In contrast, Liu et al. [34] found that DHA content was well preserved during the early development of *A. sapidissima*. This phenomenon has also been observed in other fish species [20,50], suggesting that it may be related to the specific early developmental characteristics and physiological functional differences among different fish species.

Studies have demonstrated that marine fish exhibit a selective utilization pattern of fatty acids during the prefeeding stage of larval development [39,51]. Gunasekera et al. [52], studying the early development of *Macculochella macquariensis* (trout cod), found that the order of fatty acid utilization was SFAs, MUFAs, n-6 PUFA, and n-3 PUFA. SFAs and MUFAs, as important sources of energy, were preferentially utilized, while n-3 PUFA was appropriately conserved. Han et al. [11] demonstrated that in the *S. schlegelii* larval stage, the utilization sequence of the fatty acids was MUFAs, n-6 PUFA, SFAs, and n-3 PUFA. Interestingly, the utilization rate of n-3 PUFA was the lowest, indicating its conservation. Similarly, Shi et al. [18] reported that in the *T. flavidus* larval stage, the utilization of fatty acids followed the sequence of n-3 PUFA, n-6 PUFA, SFAs, and MUFAs.

In this study, the yolk-sac larvae of pearl gentian grouper exhibited a significant decrease in SFA and MUFA contents by 24.54% and 11.68%, respectively, during the endogenous nutrition phase. Furthermore, the contents of n-3 PUFA and n-6 PUFA remained stable initially and then decreased significantly, with reductions of 8.37% and 11.62%, respectively. These findings suggest that the utilization sequence of fatty acids during the early developmental stages of hybrid grouper includes SFAs, MUFAs, n-6 PUFA, and n-3 PUFA. Moreover, numerous studies have confirmed that during the embryonic and pre-mouth-opening stages of fish during early development, SFAs and MUFAs are typically utilized as important energy sources, while n-3 PUFA is appropriately preserved. The utilization sequence of fatty acids is often observed as SFAs, MUFAs, n-6 PUFA, and n-3 PUFA [19,20,53]. These differences in fatty acid utilization patterns during the early developmental stages of fish may result from a combination of environmental factors and the energy requirements of organisms throughout long-term evolution.

## 5. Conclusions

In summary, the findings of this study indicate that there are significant decrease in total lipid, TAG, and WE-SE contents during the endogenous nutrition phase of hybrid grouper yolk-sac larvae. Essential fatty acids, such as C16:0, C18:0, C20:4n-6 (ARA), and 18:1n-9, are also notably depleted. However, the total protein content exhibits a less pronounced decrease. Among the amino acids, significant declines are observed in LEU, VAL, ISO, and PHE (EAAs), as well as in GLY, ALA, SER, PRO, and TYR (NEAAs), while the remaining amino acids show minimal changes. These results further emphasize the preferential utilization of lipids and fatty acids as crucial energy sources during the early development of hybrid grouper. Variations in the efficiency of utilizing different types of fatty acids are observed, while protein utilization remains relatively stable. The selective utilization of amino acid content is also evident, indicating specific utilization patterns during early development.

Furthermore, the sequential utilization of fatty acids during the early development of hybrid grouper is found to follow the order of SFAs, MUFAs, n-6 PUFA, and n-3 PUFA. In summary, the short duration of the endogenous nutrition period in hybrid grouper, combined with the significant increase in nutrient consumption during development, highlights the importance of providing an adequate and suitable first-feeding diet during the larval mouth-opening stage. This ensures the replenishment of essential nutrients in their bodies and enhances their survivability.

## Figures and Tables

**Table 1:** Major lipid class composition in hybrid grouper larvae at various early developmental stages (% of total lipid content on dry weight basis).

Parameter	I	II	II	IV	V
TAG	8.38 ± 0.26 ^a^	6.15 ± 62 ^b^	5.37 ± 0.15 ^c^	4.23 ± 0.18 ^d^	3.36 ± 0.22 ^e^
WE-SE	6.70 ± 0.22 ^a^	6.33 ± 0.90 ^a^	4.14 ± 0.11 ^b^	3.87 ± 0.11 ^b^	2.17 ± 0.14 ^c^
KET	3.60 ± 0.19	3.36 ± 0.31	3.58 ± 0.14	3.44 ± 0.16	3.33 ± 0.24
HC	1.62 ± 0.26	1.62 ± 0.19	1.66 ± 0.21	1.63 ± 0.11	1.66 ± 0.12
ST	18.45 ± 1.49	18.15 ± 1.19	17.76 ± 1.96	17.23 ± 1.49	16.81 ± 1.70
PL	14.97 ± 0.75 ^a^	14.63 ± 0.35 ^a^	14.07 ± 1.25 ^a^	12.06 ± 0.27 ^b^	9.35 ± 0.16 ^c^

Note: TAG, triacylglycerol; WE-SE, wax esters/steryl esters; ST, sterols; KET, ketones; HC, hydrocarbons; PL, phospholipids. I, II, III, IV, and V refer to groups for newly hatched larvae (DAH 0), 1 DAH, 2 DAH, 3 DAH, and 4 DAH, respectively. Values are expressed as the mean ± SD (*n* = 4000). Different letters indicate significant differences between groups at *p* < 0.05.

**Table 2:** Amino acid profiles of hybrid grouper larvae at various early developmental stages.

Treatment	I	II	III	IV	V
Leu	14.86 ± 1.04 ^a^	14.53 ± 0.77 ^a^	13.39 ± 1.02 ^ab^	11.76 ± 1.47 ^bc^	10.38 ± 0.93 ^c^
Lys	15.69 ± 0.09	15.20 ± 0.56	14.80 ± 0.50	14.28 ± 0.76	15.22 ± 0.43
Val	7.22 ± 0.57 ^a^	6.81 ± 0.27 ^a^	6.09 ± 0.06 ^b^	5.64 ± 0.10 ^b^	4.85 ± 0.09 ^c^
Ile	8.04 ± 0.08 ^a^	8.24 ± 0.31 ^a^	7.46 ± 0.09 ^b^	7.13 ± 0.08 ^b^	6.74 ± 0.06 ^c^
Phe	9.84 ± 0.09 ^a^	9.59 ± 0.31 ^ab^	9.30 ± 0.17 ^b^	9.14 ± 0.08 ^bc^	8.74 ± 0.17 ^c^
Met	0.48 ± 0.03	0.48 ± 0.02	0.45 ± 0.03	0.42 ± 0.03	0.43 ± 0.03
Arg	12.85 ± 1.09	12.24 ± 0.82	11.83 ± 0.09	11.38 ± 0.59	11.00 ± 0.62
His	6.30 ± 0.14	6.17 ± 0.16	6.08 ± 0.16	6.24 ± 0.07	6.04 ± 0.26
Thr	7.56 ± 0.11	7.17 ± 0.07	7.13 ± 0.42	7.18 ± 0.47	7.67 ± 0.15
EAA	74.95 ± 0.80 ^a^	72.44 ± 1.38 ^a^	69.40 ± 0.78 ^b^	66.25 ± 1.88 ^c^	62.65 ± 0.99 ^d^
Glu	20.95 ± 0.11	21.02 ± 1.31	21.00 ± 1.20	20.40 ± 0.68	19.67 ± 0.94
Asp	17.58 ± 0.33	17.19 ± 0.20	17.47 ± 0.15	17.10 ± 0.20	16.92 ± 0.44
Gly	9.92 ± 0.10 ^a^	9.73 ± 0.18 ^a^	9.29 ± 0.15 ^a^	9.03 ± 0.09 ^b^	8.92 ± 0.19 ^b^
Ala	11.52 ± 0.45 ^a^	11.14 ± 0.30 ^a^	10.10 ± 0.24 ^b^	9.16 ± 0.15 ^c^	8.44 ± 0.17 ^d^
Ser	8.67 ± 0.26 ^a^	8.51 ± 0.29 ^a^	7.76 ± 0.06 ^b^	6.95 ± 0.08 ^c^	6.22 ± 0.09 ^d^
Cys	1.27 ± 0.07	1.25 ± 0.13	1.10 ± 0.11	1.07 ± 0.12	1.11 ± 0.09
Pro	8.72 ± 0.10 ^a^	8.43 ± 0.48 ^a^	7.76 ± 0.90 ^ab^	7.22 ± 0.11 ^bc^	6.44 ± 0.10 ^c^
Tyr	7.26 ± 0.05 ^a^	7.27 ± 0.20 ^a^	7.08 ± 0.12 ^ab^	6.88 ± 0.17 ^b^	6.87 ± 0.08 ^b^
NEAAs	85.96 ± 0.81 ^a^	83.35 ± 1.63 ^ab^	81.42 ± 1.47 ^bc^	77.82 ± 0.73 ^c^	74.55 ± 1.52 ^d^
EAAs/NEAAs	0.87 ± 0.01	0.87 ± 0.03	0.85 ± 0.02	0.85 ± 0.02	0.84 ± 0.01
TAAs	160.92 ± 1.14 ^a^	155.79 ± 1.66 ^b^	150.82 ± 1.24 ^c^	144.07 ± 2.40 ^d^	137.20 ± 2.33 ^e^

Notes: Data are presented as the mean ± S.D. (*n* = 4000). Values with different letters denote significant differences among treatments (one-way ANOVA, *p* < 0.05). Amino acid contents (μg larvae^−1^); I, II, III, IV, and V refer to groups for newly hatched larvae (DAH 0), 1 DAH, 2 DAH, 3 DAH, and 4 DAH, respectively. Asp, aspartic acid; Ser, serine; Glu, glutamic acid; Gly, glycine; Ala, alanine; Cys, cysteine; Pro, proline; Tyr, tyrosine; Val, valine; Met, methionine; Ile, isoleucine; Leu, leucine; Thr, threonine; Phe, phenylalanine; Lys, lysine; His, histidine; Arg, arginine; EAA, essential amino acid; NEAA, non-essential amino acid; TAA, total amino acid.

**Table 3:** Fatty acid composition of hybrid grouper larvae at different early developmental stages (% of total fatty acids).

Fatty Acid Type	I	II	III	IV	V
C12:0	0.14 ± 0.01	0.15 ± 0.01	0.14 ± 0.01	0.14 ± 0.02	0.15 ± 0.01
C14:0	0.77 ± 0.03	0.74 ± 0.05	0.77 ± 0.04	0.77 ± 0.05	0.76 ± 0.05
C16:0	27.27 ± 0.61 ^a^	26.87 ± 0.60 ^a^	25.27 ± 0.47 ^b^	22.78 ± 0.64 ^c^	20.37 ± 0.55 ^d^
C18:0	6.71 ± 0.16 ^a^	6.47 ± 0.26 ^a^	5.93 ± 0.09 ^b^	5.62 ± 0.11 ^c^	4.91 ± 0.15 ^d^
C20:0	0.61 ± 0.02	0.60 ± 0.02	0.63 ± 0.01	0.61 ± 0.02	0.61 ± 0.04
∑SFA	35.50 ± 0.04 ^a^	34.84 ± 0.80 ^a^	32.73 ± 0.53 ^b^	29.93 ± 0.49 ^c^	26.79 ± 0.48 ^d^
C16:1	0.38 ± 0.21	0.33 ± 0.04	0.36 ± 0.03	0.35 ± 0.04	0.36 ± 0.02
C16:1n-7	1.56 ± 0.11	1.64 ± 0.10	1.60 ± 0.13	1.74 ± 0.09	1.72 ± 0.12
C18:1n-9	22.17 ± 0.49 ^a^	21.16 ± 0.35 ^b^	20.29 ± 0.57 ^c^	19.08 ± 0.22 ^d^	18.28 ± 0.25 ^e^
C18:1n-7	2.56 ± 0.12	2.71 ± 0.16	2.58 ± 0.14	2.57 ± 0.22	2.62 ± 0.19
C20:1n-9	1.80 ± 0.06	1.77 ± 0.10	1.74 ± 0.09	1.78 ± 0.10	1.73 ± 0.06
C20:1n-7	0.42 ± 0.07	0.46 ± 0.06	0.44 ± 0.05	0.47 ± 0.03	0.45 ± 0.03
C22:1n-9	2.08 ± 0.04	2.12 ± 0.22	2.11 ± 0.14	2.17 ± 0.25	2.19 ± 0.12
∑MUFA	30.98 ± 0.51 ^a^	30.19 ± 0.09 ^a^	29.11 ± 0.57 ^b^	28.16 ± 0.51 ^c^	27.36 ± 0.43 ^c^
C18:2n-6	2.95 ± 0.10	2.78 ± 0.16	2.92 ± 0.15	2.97 ± 0.12	2.90 ± 0.24
C18:3n-6	0.31 ± 0.02	0.33 ± 0.06	0.35 ± 0.04	0.35 ± 0.03	0.34 ± 0.04
C18:3n-3	2.25 ± 0.10	2.46 ± 0.08	2.95 ± 0.12	3.32 ± 0.10	3.65 ± 0.17
C18:4n-3	1.03 ± 0.09	1.05 ± 0.11	0.97 ± 0.18	1.02 ± 0.13	1.04 ± 0.11
C20:3n-6	0.79 ± 0.08	0.83 ± 0.10	0.83 ± 0.13	0.85 ± 0.12	0.87 ± 0.06
C20:3n-3	0.08 ± 0.01	0.08 ± 0.01	0.08 ± 0.01	0.08 ± 0.01	0.08 ± 0.01
C20:4n-6	2.32 ± 0.03 ^a^	2.22 ± 0.18 ^a^	2.19 ± 0.22 ^a^	1.89 ± 0.08 ^b^	1.51 ± 0.04 ^b^
C20:5n-3	4.51 ± 0.16	4.28 ± 0.27	4.48 ± 0.33	4.50 ± 0.13	4.51 ± 0.24
C22:5n-3	1.63 ± 0.14	1.90 ± 0.15	1.93 ± 0.15	1.76 ± 0.12	1.89 ± 0.16
C22:6n-3	27.28 ± 0.29 ^a^	27.04 ± 0.07 ^a^	27.11 ± 0.51 ^a^	26.47 ± 0.15 ^b^	22.54 ± 0.01 ^b^
∑PUFA	43.16 ± 0.23 ^a^	42.97 ± 0.12 ^a^	43.82 ± 0.95 ^a^	43.21 ± 0.26 ^a^	39.34 ± 0.34 ^b^
n-3	36.79 ± 0.24 ^a^	36.81 ± 0.40 ^a^	37.52 ± 0.82 ^a^	37.15 ± 0.30 ^a^	33.71 ± 0.23 ^b^
n-6	6.37 ± 0.16 ^a^	6.16 ± 0.38 ^a^	6.30 ± 0.15 ^a^	6.06 ± 0.08 ^a^	5.63 ± 0.29 ^b^
n-3/n-6	5.78 ± 0.16	5.99 ± 0.45	5.96 ± 0.07	6.13 ± 0.11	6.00 ± 0.32
DHA/EPA	6.05 ± 0.21 ^a^	6.34 ± 0.40 ^a^	6.07 ± 0.36 ^a^	5.88 ± 0.15 ^a^	5.00 ± 0.27 ^b^
EPA/ARA	1.95 ± 0.05 ^a^	1.94 ± 0.27 ^a^	2.06 ± 0.34 ^ab^	2.39 ± 0.17 ^b^	2.98 ± 0.12 ^c^
DHA+EPA	31.79 ± 0.35 ^a^	31.32 ± 0.31 ^a^	31.59 ± 0.78 ^a^	30.97 ± 0.23 ^a^	27.05 ± 0.23 ^b^

Notes: Data are presented as the mean ± SD. (*n* = 4000). Values with a different letter denote significant differences among treatments (one-way ANOVA, *p* < 0.05). I, II, III, IV, and V refer to groups for newly hatched larvae (DAH 0), 1 DAH, 2 DAH, 3 DAH, and 4 DAH, respectively. SFA, saturated fatty acid; MUFA, monounsaturated fatty acid; PUFA, polyunsaturated fatty acid; n-6, n-6 polyunsaturated fatty acids; n-3, n-3 polyunsaturated fatty acids; EPA, eicosapentaenoic acid (20:5n-3); DHA, docosahexaenoic acid (22:6n-3); ARA, arachidonic acid (20:4n-6).

## Data Availability

The data that support the findings of this study are openly available on Figshare at https://figshare.com/s/dc08ddd7fd4a9b01fb54, doi.org/10.6084/m9.figshare.23957943.
